# Catalyzing Neurophysiology: Jacques Loeb, the *Stazione Zoologica di Napoli*, and a Growing Network of Brain Scientists, 1900s–1930s

**DOI:** 10.3389/fnana.2019.00032

**Published:** 2019-03-18

**Authors:** Frank W. Stahnisch

**Affiliations:** Alberta Medical Foundation/Hannah Professor in the History of Medicine and Health Care, University of Calgary, Calgary, AB, Canada

**Keywords:** history of neuroanatomy, Jacques Loeb, Germany, Italy, United States, 20th cent. history of medicine, regeneration, brain research

## Abstract

Even before the completion of his medical studies at the universities of Berlin, Munich, and Strasburg, as well as his M.D.-graduation – in 1884 – under Friedrich Goltz (1834–1902), experimental biologist Jacques Loeb (1859–1924) became interested in degenerative and regenerative problems of brain anatomy and general problems of neurophysiology. It can be supposed that he addressed these questions out of a growing dissatisfaction with leading perceptions about cerebral localization, as they had been advocated by the Berlin experimental neurophysiologists at the time. Instead, he followed Goltz and later Gustav Theodor Fechner (1801–1887) in elaborating a dynamic model of brain functioning *vis-à-vis* human perception and coordinated motion. To further pursue his scientific aims, Loeb moved to the Naples Zoological Station between 1889 and 1890, where he conducted a row of experimental series on regenerative phenomena in sea animals. He deeply admired the Italian marine research station for its overwhelming scientific liberalism along with the provision of considerable technical and intellectual support. In Naples, Loeb hoped to advance his research investigations on ‘tropisms’ further to develop a reliable basis not only regarding the behavior of lower animals, but also concerning perception and general neural capacities. He thought that he could demonstrate the existence of center interdependence in the cerebral cortex of higher animals and humans, and was convinced that regenerative phenomena existed as plastic mechanisms influencing animal as well as human behavioral qualities. This new perspective on the organization of brain functioning and Loeb’s astonishing successes in experimental research with hydrozoa and echinoidea brought him in close contact with many biologists working on the nervous system during the early twentieth century. Yet, it is impossible to conceive of Loeb’s ground-breaking experiments without also taking his contemporary scientific network of teachers, colleagues, and local research milieus into account. All of these exerted a strong influence on a growing network of physiology, anatomy, and neurology peers and research trainees, who went on to interact in early brain research centers in Central Europe and North America. This article explores some intellectual and organizational influences that developed out of Loeb’s early experiences at the Naples Zoological Station in Italy. The main focus is laid here on questions of the structure and organization of scientific institutions, the development of research networks among biologists of the nervous system, as well as the emergence of an interdisciplinary research style during the early decades of the twentieth century. This innovative style of laboratory investigations later influenced the make-up of a number of research units, for example at the Kaiser Wilhelm Society in Germany and the Rockefeller Institute for Medical Research in the United States.

## Introduction

**FIGURE 1:**
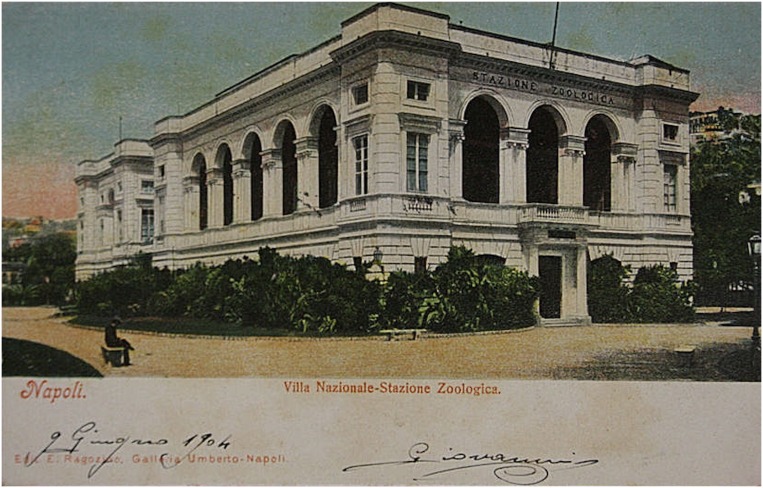
*Stazione Zoologica di Napoli* (ca. 1910). Photograph © Public Domain.

Through focusing on a particular historical episode of the Naples marine research station (Italian: “*Stazione Zoologica di Napoli*”) in this article, I aim at examining a remarkably fruitful period of international relations and disciplinary interactions at the beginning of the twentieth century. This foremost biological research institution on the Mediterranean Sea attracted many outstanding scientists and postgraduate students from a new generation of experimental biologists, neurophysiologists, and laboratory-based neurologists to visit Italy in Southern Europe (Deichmann, 2010). Nevertheless, at the very center of my narrative is the working life and training experience of an individual laboratory researcher, *viz.* the influential German-American experimental biologist Jacques Loeb. After Loeb had first been disappointed not to receive a working place at the marine biological station in 1888, because all places for visiting scientists were then occupied, he could pay visit to the Naples marine research station throughout a two-year period from 1889 (Oct-10 to May-1) to 1890 (Oct-31 to April-25) and later returned on multiple occasions to Italy for shorter follow-up stays (Fangerau, 2014; Sgambato et al., 2016). He undertook his laboratory research in Naples out of a decided interest to experience first-hand the promising laboratory conditions at the seaside (Figure 1), of which he heard through returning German biomedical scientists who had made the trip earlier (Breidbach, 1997).

One of those laboratory veterans, who had earlier traveled to Italy, was Loeb’s own anatomy professor at Strasburg’s Kaiser Wilhelm University, the specialist for morphological brain research Gustav Schwalbe (1844–1916). Schwalbe was himself a student of Germany’s most prominent Darwinian, the developmental biologist Ernst Haeckel (1834–1919). The latter’s relationship with the marine research station in Naples has been researched more widely in scholarly literature (Olsson et al., 2009). Although Loeb later emerged as a general experimental biologist – working on organismic adaptation, cell communication, growth development, and nutritional processes (Loeb, 1918a) – his particular research interest in brain and spinal cord problems emerged from his earlier medical studies, before he graduated as a trained physician during the times of Imperial Germany (Osterhout, 1928).

This article initially examines Loeb’s formative research interests in degenerative and regenerative phenomena primarily in invertebrate animal models (Loeb, 1892), along with his contributions to the experimental neurology of memory, functional localization, and neuronal plasticity. A succinct outline of his own expectations prior to traveling to Italy is provided in line with his personal experiences during the laboratory research-stay at the marine research station in Naples. In the final part, a growing network of Loeb’s collaborators and peers in modern biology of the nervous system from 1910 to 1930 is examined. This organizational setting has long been neglected in historiography despite its vital importance for Loeb’s working and professional development, likely because historical research has primarily focused on Loeb’s more general contributions to experimental biology and developmental genetics (e.g., Rasmussen and Tilman, 1998) as well as persisting research biases on later North American analogs of the *Stazione Zoologica di Napoli* (such as in Kohler, 2002), which neglected the important precursor institutions and thus foundational developments since the nineteenth century in Europe.

## Jacques Loeb as an Early Brain Scientist

**FIGURE 2:**
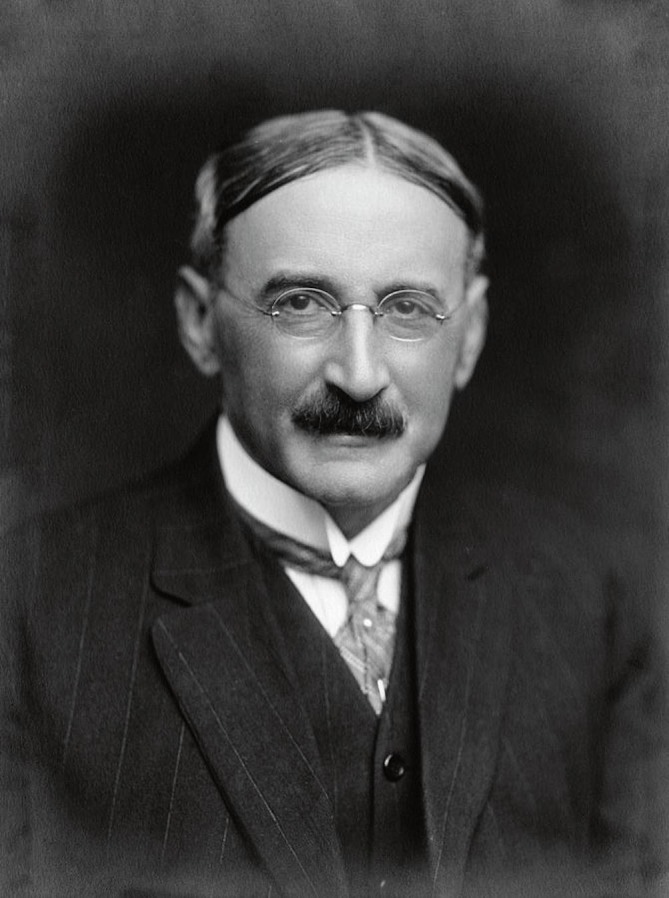
Portrait photograph of Jacques Loeb. © Chemical Heritage Foundation in Philadelphia, PA, United States.

Isaak Jacques Loeb was born in 1859 in Mayen in the Rhineland, not far from the Friedrich Wilhelms University of Bonn, into an affluent retail merchant family (Figure 2). His father had been a tradesman with strong social and commercial contacts to France, who introduced his son to a wealth of French ideas and formidable literature (Downes, 1924). Yet he lost both his parents early during his adolescence, and it was decided that he should be raised by his uncle in the Prussian capital of Berlin, where he also worked in his uncle’s private bank to contribute to the cost of his education (Rasmussen and Tilman, 1998). After his graduation from the grammar school Ascanisches Gymnasium, he sat out to study philosophy from 1880 onward with the idealist philosopher Friedrich Paulsen (1846–1908) at Berlin’s Friedrich Wilhelms University. Although Loeb had by then developed a marked interest in the area of philosophy of mind and the phenomenology of Ernst Mach (1838–1916) (Heidelberger, 2010), he nevertheless decided to turn to more practical work and study medicine at the Universities of Berlin, Munich, and Strasburg.

In 1884 at the research-intensive *milieu* of Strasburg’s newly founded Kaiser Wilhelms University, Loeb graduated with an M.D. under the supervision of Friedrich Goltz (1834–1902) as the *ordinarius* professor of experimental physiology. Only 1 year later, Loeb also received his medical license to practice. At the Alsacian university, Goltz had successfully nurtured his interest in brain research topics to such a degree that another one of Goltz’s trainees, Dr. Anton Nessler (d. 1917?) – a licensed physician from Wehingen in Wuerttemberg in southwest Germany –, cited Loeb four times in his “*Injuries of the Occipital Lobes of the Brain and their Resulting Disturbances*,” basing his thesis strongly on Loeb’s foregoing work (Nessler, 1891).

Hereafter, Loeb left Strasburg for three consecutive years for a European peregrination to visit with the German physiologist Nathan Zuntz (1847–1920). Zuntz acted as a training mentor to Loeb and successfully advocated his work to other physiologists, chemists, and medical scientists (see Loeb, 1908), after the latter joined Zuntz at the Agricultural College of Berlin in 1885. When returning to Goltz’s physiology laboratory in Strasburg, as the second research assistant to the institute (“*2. Assistent*”) from winter semester 1888 to the summer semester 1890 (Bonah, 2006), Loeb became further interested in scientific issues of nervous degeneration and regeneration.

After he became an independent researcher through his own standing, Loeb additionally broadened his experimental work toward neuroplasticity topics, including for example investigations on peripheral nerve injuries from both neurosurgical and neuropathological perspectives. He based these visibly on mechanistic and physico-chemical interpretations, as he later intriguingly summarized.

“We can, therefore, state that the quantity of regeneration in an isolated piece of an organism is under equal conditions and in equal time directly proportional to the mass of growth material circulating in the sap (or blood) of the piece and required for the synthetical processes giving rise to the regenerated tissues and organs. If we measure the rate of regeneration by the mass of material regenerated in a given time, the law expressed for the quantity holds also for the *rate* of regeneration and in this form the law becomes a special case of the law of chemical mass action” (Loeb, 1918b, emphasis in the original).

Manifestly, the medical experience of long-term effects from the Franco-Prussian War of 1870/1871 left a noticeable impact on physicians and medical scientists on both sides of the Rhine. Knowledge regarding peripheral and central forms of neurosurgery, wound treatment, and neurorehabilitation (Stahnisch and Nitsch, 2002) was then desperately needed to care for the thousands of war-injured soldiers returning from the French frontlines. Having been recruited as a research assistant by Goltz in 1888 for his physiological department, Loeb became responsible for the laboratory organization and teaching courses for the medical students. Through Goltz’ well-known research with cortical ablations in dogs and other animals, to elicit functional motor loss, improved understanding of the mechanisms involved in visual perception and speech formation, Loeb was also introduced to broader de- and regeneration problems in contemporary brain research (Stahnisch, 2009). Loeb’s own experimental research tried to find particular answers to the so-called “localization problem,” related to the capacity of distinct parts of the brain to morphologically adapt to experimental lesions (Finger, 2005).

As emanates from the Strasburg academic register of doctoral theses, diploma certificates, and *Habilitationsschriften* (Archives Départementales de Strasbourg, 1891), Goltz was very fond of his pupil’s experimental work. Moreover, published seminal works of Loeb (e.g., Loeb, 1884) were placed next to translations of Pierre Flourens’ (1794–1864) seminal publications, and the ablation studies of Hermann Munk (1830–1912) and David Ferrier (1843–1928) in “*Pflueger’s Archiv fuer die gesamte Physiologie des Menschen und der Tiere*” (“*Pflueger’s Archive for General Human and Animal Physiology*”). While rising as a skilled experimental biologist at the time, it is nevertheless important to keep in mind that Loeb addressed these questions out of a wider philosophical interest than Goltz (Loeb, 1885), placing his psychological concept to analyze the workings of the mind in a decidedly modular context of various brain systems (Loeb, 1886). With respect to his own laboratory approaches, Loeb remained quite dissatisfied with leading perceptions of cerebral localization as advocated by the Berlin experimental physiologists Emil Heinrich DuBois-Reymond (1818–1896) and Hermann Munk, who had advocated for specific functional centers and unidimensional functional losses that would follow each brain injury (Benton, 2000). Instead, Loeb sided with his mentor Goltz and the late experimental psychologist Gustav Theodor Fechner (1801–1887) at the University of Leipzig in their development of a dynamic model of brain physiology and functional motor coordination (Loeb, 1906a).

The formative Strasburg period of Loeb and his indebtedness to his teacher Friedrich Leopold Goltz are regrettably understudied; and this concerns also Loeb’s early interests in neuronal regeneration research growing out of the Strasburg context (Stahnisch, 2016). As one can apprehend from his early experimental approaches, as historian Philipp J. Pauly has shown in his insightful study “*Controlling Life – Jacques Loeb and the Engineering Ideal in* Biology” (1987) (Pauly, 1987), Loeb developed a medically oriented approach to the problem of regeneration and functional gain in the human nervous system. These views were visibly opposed to German zoologist and experimental biologist Wilhelm Roux’s (1850–1924) developmental mechanics (“*Entwicklungsmechanik*”) (Roux, 1964), which Loeb regarded as too rigid for adequate biological explanations (Roux, 1905):

“Regeneration is the re-establishment of amputated limbs and other thoroughly developed parts of the body that have been lost, i.e., it is a restitution process. […] Regeneration is brought about mechanically, after Roux [he talks of himself in the third person], because the cells of the fully developed body entail somatic germ plasma […]. And the particular kind of defect brings about the necessary supplementation from this omnipotent [biological] stock” (Roux, 1888).

Pauly has drawn a direct line from Loeb’s working context in Strasburg to his subsequent career in the United States, such as at Berkeley and the University of Chicago, where he became a professor of physiology between 1892 and 1902. When working in Chicago, the Hull Biological Laboratories forged a synergistic conglomerate of research buildings together with the zoology and anatomy departments (Lazar, 2018). These buildings would comprise the zoological department, chaired by Charles Otis Whitman (1842-1910), the botanical laboratory chaired by John Merie Coulter (1851–1928), Jacques Loeb’s own physiology chair, and the anatomical laboratory headed by Henry Herbert Donaldson (1857–1938) (Henry and Collins, 1902). From Chicago, in 1910, Loeb then went to the Rockefeller Institute for Medical Research in New York City, which enabled extended visits to the nearby Marine Biological Laboratory in Woods Hole in Massachusetts. Woods Hole, for him, was a rather rustic institution – an ensemble of “gingerbread” cottages with a small laboratory building – that always granted important access to experimental sea animals. Already since 1904 he had considered a new organizational model for experimental laboratory research, which he described in his letter to American biologist and eugenicist Charles Davenport (1866–1944) on April 4 of that year in the following words:

“There is some talk of putting up a station here [in the United States] and I have been asked to furnish some suggestions. Do you possess a set of plans of the Station at Naples and if so would you be kind enough to let me have them for a few days?” (Loeb, 1904 cit. after Fangerau, 2014).

During his summer breaks there, Loeb advanced his “engineering standpoint” further toward basic neurology and experimental biology (Loeb, 1912b), based on his developmental work with sea urchins and tubularia, while crossing embryological with traumatological paradigms with the first stages of embryonic development. Loeb visited Woods Hole numerous times from New York and used his contacts actively to acquire philanthropic funding for the marine research station. Through his academic acquisitions, he increased its international visibility by inviting German-American experimental neurophysiologists, such as neurochemist and synapse researcher Otto Loewi (1873–1961) (Loewi, 1921) or nerve sheath investigator Martin Silberberg (1895–1969), to join neuroregenerative laboratory studies (Loeb, 1924). Yet, Loeb’s understanding of the neuronal mechanisms of degeneration and regeneration was quite different, in that he addressed basic control phenomena in living organisms first by means of physiological experimentation and later through medical approaches to functional restitution. Through his pursuit, he famously shaped a whole generation of American physiologists, introducing them to his “engineering ideal” of developmental biology (Loeb, 1922). Among this group counted, for example, German émigré researcher Richard Benedict Goldschmidt (1878–1958) at the University of California, Berkeley (Goldschmidt, 1924), as well as John Howard Northrop (1891–1987) at New York’s Rockefeller Institute (Stahnisch, 2006).

In the following, I try to show how Loeb’s mechanistic engineering approach of living processes and the functional organization of the brain became later introduced into the early experimental biology field of the nervous system. Thus, his statement “finally, we can control the most fundamental processes of life” (Loeb, 1899) was gradually taken up by prominent comparative neurologists and neuroanatomists of the time, who showed an active interest in neuroregenerative phenomena, *viz.* neurologist Ludwig Edinger (1855–1915) and neurophysiologist Albrecht Bethe (1872–1954) in Germany (Fischer, 1957), or American neuroanatomist Henry H. Donaldson (1857–1938) of the Wistar Institute in Philadelphia (Stahnisch, 2003b). Having situated Loeb’s research influence in the experimental brain science community of the time, it is indispensable to review the preceding experiences which Loeb had made as a visiting scientist at the marine research station in Naples and to see how these observations fostered his aims for building a medically inspired “technical biology” (Fangerau, 2009).

## The *Stazione Zoologica Di Napoli*

After Loeb embraced engineering mechanisms as his ultimate research goal in experimental biology, he moved south to Italy from Strasburg for a two-year-long research visit from 1889 to 1890. The Naples marine research station had already been established in 1870 by Anton Felix Dohrn (1840–1909), a young Prussian zoologist. The idea to build this institution had come up to Dohrn during an encounter with Russian ethnographer and biologist Nicholas Miklouho-Maclay (1846–1888) in Messina, Sicily, where the two scientists had met and struck a plan to build a globally connected network of zoological research stations (Groeben, 1985). Their model was taken from the ever-growing network of railway stations, easily accessible and well-supported, facilitating fast interchanges and face-to-face communication. Experimenters and observers could easily move from one biological station to the next, find adequate places of accommodation, receive the experimental models and tools needed for their work – and be independent of local infrastructures, laboratories, and personnel at their home institutions (Groeben, 2006).

Dohrn had initially used his own wealth and later personal contacts to the Kaiser Wilhelm Society, in particular to inaugural president Adolf von Harnack (1851–1930) (Max Planck Society Archives, 1930), to facilitate the build-up and organization of the marine research station in Naples. Various relations between German and Italian science elites and associations furthered the construction of an extensive research building and public aquarium at the Bay of Naples (Barnardino, 2000). As director of the station, Dohrn lived an almost idyllic life. It combined the style of genteel research in morphology with a continuous flow of stimulating guests from all over the world (Dohrn, 1930). Other international visitors included for example Berlin neurologist Friedrich Heinrich Levy (1885–1950), who – in 1914 – had worked with Ludwig Edinger in Frankfurt am Main. Levy later needed to emigrate from Nazi Germany to Philadelphia, finding work and a new living context in America, similar to émigré scientists as Loeb himself, who had left Germany prior to 1933 (Russell and Stahnisch, 2017). At Dohrn’s celebrated marine research station in Naples, Lewy (who had changed the spelling of his name upon naturalization as a US citizen) did much scientific fieldwork and basic physiological experiments, then quite uncommon in experimental neurology (Groeben and de Sio, 2006). Equally, German-British experimental neurologist and neuroanatomist Paul Glees (1909–1999) joined the biological center, having collaborated with Dutch neuroanatomist Cornelius Ubbo Ariëns Kappers (1877–1946), the director of the comparative-anatomical department of the “*Nederlands Instituut voor Hersenonderzoek*” (“*The Brain Research Institute of The Netherlands*”), before. In Amsterdam, Glees had acquired a broader zoological understanding of comparative neurology, than many contemporaries possessed, which served him and his associates in Naples very well.

Already Dohrn had acquired a solid scientific standing for the Naples marine station, nurtured through his international correspondence with Charles Judson Herrick (1858–1904) at Clark University and his and his co-workers’ repeated submissions of articles to the latter’s *Journal of Comparative Neurology* in the United States (Bartelemez, 1973). Since his earlier time as a zoologist, Dohrn himself had been an ardent experimenter and developmental morphologist, studying the origins of species and topics of evolution from a common ancestor to understand principles of functional change due to the structural set-up of biological variance (Dohrn, 2007). In the following, Dohrn’s marine research station came to offer a platform for multiple comparative programs analyzing the nervous system from anatomical and physiological perspectives, about which Berlin experimental physiologist Emil Du-Bois Reymond noted in an early letter to Dohrn, in 1870, that Naples represented “an aquarium supplemented by a physiological laboratory.” In the following decades, the marine research station attracted hundreds of scientists, including Jacques Loeb (see Jahn, 2000), who needed to return and revisit Naples’ rich sea life during the breeding seasons of the experimental sea animals.

Traveling swiftly from Germany to southern Italy by railway, he could easily get to the marine research station, plan and conduct various experimental series on regenerative phenomena in sea animals, and profit from the excellent surroundings and social amenities. He deeply admired the Naples marine research station for its overwhelming liberalism as well as its technical support that it offered to each visiting scientist.

**FIGURE 3:**
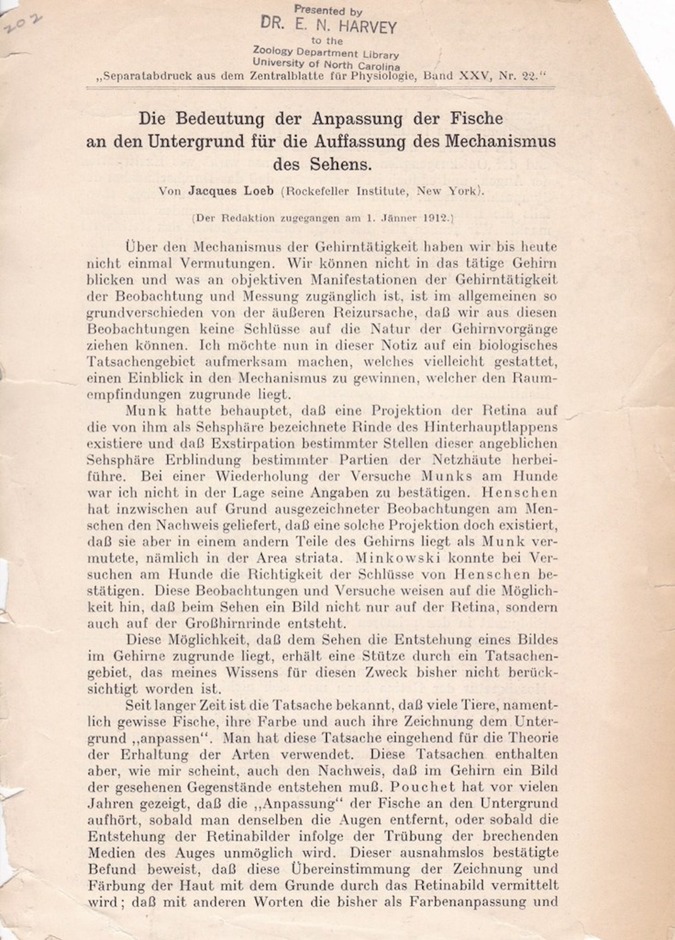
Cover page of Loeb (1912a). Die Bedeutung der Anpassung der Fische an den Untergrund fuer die Auffassung des Mechanismus des Sehens. *Zentralbl. f. Physiol.* 15, 22, 1015–1019.

Even if the Naples marine research station did not have a close connection to the Italian universities at the time, it represented a marvelous research paradise for experimental biologists and medical scientists, offering vivid exchanges of methods and experiences to conduct innovative laboratory research. Compared with the limited variety of species in the Baltic Sea – that Loeb had seen before that time – he became immensely impressed with the opulence of biological species from the Mediterranean Sea (Figure 3) in the Bay of Naples – spanning from Ischia and Cape Miseno west of the city to the Island of Capri, Sorrento and Positano south of the bay.

He found a much richer quantity of all-new creatures, accessible through diving with the station’s new diving helmet as well as sampling on the beaches, to fill the large aquaria of the marine research station (Simon, 1980). In Naples, Loeb could concentrate on his experiments alone and did not need to consider specific time or space requirements for his experiments – just following through with his hypotheses in an optimal research program. He was seen to have scuba-dived twice a week and spent all the mornings at his work place, to experiment with fish (Figure 3), hydracea, muscles, jelly fish, and sea urchins. The afternoons and evenings he appeared to have used for entertainment at local restaurants – or writing manuscripts, sending these off to his publishers in Germany, Italy, France, and America, along with other types of personal correspondence. On occasion, Loeb delivered lectures at the marine research station itself and to regional natural history or physicians’ associations.

In Naples, he also engaged in closer academic and personal contacts with American scientists, such as William W. Norman (d. 1898), an associate professor of biology from the University of Texas, and Thomas T. (“Percy”) Groom (1876–1942), a recent Cambridge University zoology graduate, who developed a continuing career in the United States (Texas Academy of Science, 1945). Loeb’s own reasons for first emigrating to the elite Quaker Bryn Mawr College for female students in the United States, however, were a deeply felt result of the social hierarchies in the academy of his native Germany. It was difficult for Jews to find employment in mainstream scientific disciplines, and it proved even more challenging to be an advocate of a newly emerging one, such as experimental zoology/comparative neuroanatomy (Ringer, 1969). Loeb’s liberal political attitude predisposed him to criticisms from the obstinate Prussian administrative *status quo* in the recently formed Empire under Kaiser Wilhelm I (1797–1888) (Rasmussen and Tilman, 1998). Yet the following year also held a pleasant personal kismet for him:

“In the spring of 1890, at the home of Professor Justus Gaule [1849–1939] (professor of physiology in Zurich and a former assistant of Goltz), he met a young American, Miss Anne Leonard [1862–1951], who had just received her doctorate in philology at the University of Zurich. The acquaintance resulted in an engagement and they were married in October of the same year. After the marriage, which took place in America, they returned to Naples multiple times […]. There, he devoted himself to experiments on heteromorphosis since he was convinced that not only the “will” of the animal but also the form and function of its organs and its course of development might be controlled by the experimenter, an idea quite contrary to concepts then prevailing” (Osterhout, 1928).

One of these return visits occurred in 1911. This extended research stay was made possible through a payment of 1,000 Reichsmark for his personal uses by the development company advancing the creation of the German Kaiser Wilhelm Society (Max Planck Society Archives, 1911). As has been pointed out by German medical historian Heiner Fangerau, early regenerative investigations undertaken by Loeb himself addressed their questions through a type of “wild experimentation” at the Naples Zoological Station:

“With an exception of those few individuals [experimental animals], which recently fell into the hands of those physiologists who worked on galvanotropism, no animal could be conceived to be put under electric currents. Yet in reality, galvanotropism is a rather extraordinary widespread phenomenon among the animal kingdom. There is no stronger contradiction than those criticisms, that animals’ reactions would not be determined by their biological needs and interests, nor acquired through the process of natural selection […]” (Loeb, 1909).

Loeb found it much more comfortable to work with experimental animals such as hydroids, actinia, and even sharks, rather than using dogs as in Strasburg before.

**FIGURE 4:**
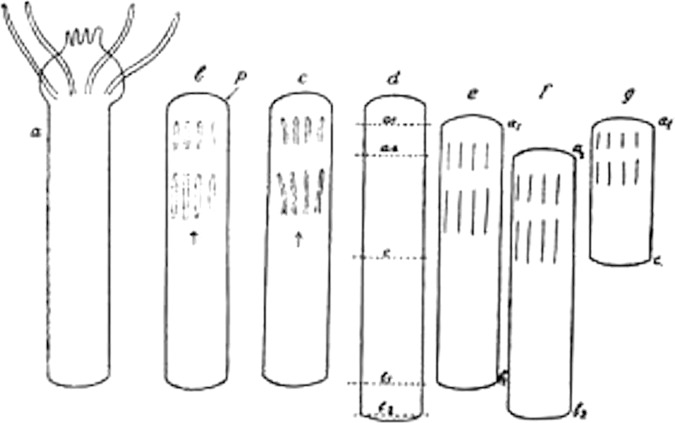
Jacques Loeb’s widely received *Tubularia* dissection and regeneration experiments (1921). Ink drawing © Public Domain.

The physiological functions of Mediterranean Sea animals were more transparent and easily addressable than those of the terrestrial organisms he had experimented with before (Fangerau, 2014). As such, successful research in neurophysiology and neuroanatomy at the time was also represented by access to the right object in the laboratory. While conducting his investigations of regenerative phenomena, Loeb – who came to favor the physiology of hydroids such as tubularia – found that a free floating tubularia stem of particular length would not produce a new body (hydrant) at its distal end (Loeb, 1904; Figure 4).

He rather formed a secondary incision at its dorsal end. From isolated fragments of plantaria, in 1891, Loeb was able to produce animals with two heads and primitive central nervous systems, a phenomenon for which he coined the term “heteromorphosis (Maienschein, 1994).”

“I have succeeded in finding animals in which it is possible to produce at desire a head in the place of a foot at the aboral end, without injuring the vitality of the animal. […] A Tubularian has by artificial means been so altered that it terminates in a head at both its oral and aboral ends. If, for any reason, it was necessary to create any number of such bioral Tubularians, this demand could be satisfied” (Loeb, 1891).

He saw himself in the revolutionary role of being a biomedical engineer, who had discovered that systematic experimental changes in the body led to morphologically altered ontogenetic structures. This assumption was not without consequences for his own physiological interpretations of nerve center functioning either, since he developed the firm opinion that neuronal regenerative capacities existed as general mechanisms of plasticity following nervous system injury in animals and humans.

This new perspective and Loeb’s astonishing experimental research on hydroids brought him into close exchanges with leading American experimental biologists, such as chemico-physical tropism specialist, Thomas Hunt Morgan (1866–1945) – who received the Nobel prize for Physiology or Medicine in 1933 – Yale anatomist Ross Granville Harrison (1870–1959), James McKeen Cattell (1860–1944) from the University of Pennsylvania, and Winthrop John Van Leuven Osterhout (1871–1964) at Harvard University. They remained Loeb’s collaborators and professional friends along with a good number of early biologists working on the nervous system, as shall be examined in the next part of this article (Andersen, 2004).

## A Growing Network of Brain Scientists During the Early Twentieth Century

Soon after Loeb’s death, well-known biologist Curt Herbst (1866–1946) had described how exciting Loeb’s “*Untersuchungen zur physiologischen Morphologie der Thiere*” (“*Investigations on Animal Physiological Morphology*,” 1891) had been for a young generation of experimental zoologists (Loeb, 1891). According to Herbst, Loeb not only threw “light like a bright sunbeam into the darkness of morphology,” yet he had also started a new wave of experimental research on neuronal regeneration (Herbst, 1924). It is, however, impossible to conceive of Loeb’s ground-breaking experimental series without taking his wider scientific network of teachers, colleagues, and local research *milieus* into account. They exerted a remarkable influence on the ever-growing number of his collaborators, neurophysiological peers, and research pupils.

I now want to look closer at those intellectual and organizational influences that developed out of Loeb’s early experiences from the marine research station. For this aim, if we refer to the sociological Actor-Network Theory – as it has been developed by French scholars Bruno Latour and Michel Callón (Callón and Latour, 1981) – we can see how also in the case of Loeb’s experimental biology wider intellectual interests had been at play, which complemented and cross-fertilized each other. These included previous stimuli from the philosophy of mind, particularly German Idealist positions (as in Ernst Mach’s philosophy of science), and later also from behaviorism – with American psychologist James B. Watson (1878–1958) being among Loeb’s own research trainees. Other historical actors included regional scientific societies as well as accessibility to required technological resources (aquaria, dissection tables, experimental animals, microscopes, staining technologies, and pathological specimens for comparative collections). Although the Naples marine research station had also been an important location for the first application of the cinematographic method by the French neurophysiologist Étienne-Jules Marey (1830–1904) and the Italian neurophysiologist Osvaldo Polimanti (1869–1947) (Sedda, 2012), who used tachistographic photo-devices for their studies on neuromuscular interactions in movement, no primary evidence could be found in the archival materials and publications of Loeb, that he would have used cinematography already in Naples himself. Yet generally, cutting edge technologies served as vital ingredients of Loeb’s laboratory-based research program, particularly after he had become a founding member of the *Journal of General Physiology* (Fangerau, 2014). Sociologists Latour and Callón regard such scientific spheres and multiple historical actors as closely related regarding their locality and complexity, scientific activities, and as epistemic catalyzers – here of basic research on the nervous system:

“ANT [Actor-Network-Theory] furnishes us with the tools to *better* attend to the minute displacements, translations, practices, riots, processes, protests, arguments, expeditions, struggles, and swap-meets – no matter what the actors involved may look like. Crucially this is work and labor that might otherwise have been neglected in analysis and yet is absolutely essential to understand the inner-workings of our collectives. It is for this reason that ANT aims to become insensitive to any *a priori* difference between humans and non-humans. The perspective asks that we remain open to the possibility that non-humans add something that is of sociological relevance to a chain of events: that something happens, that this something is added by a non-human, and that this addition falls under the general rubric of action and agency. It is the action itself that is the important thing to trace” (Callón and Latour, 1981, emphasis in the original).

So, with the ANT perspective, we can furbish fuller explanations of Loeb’s scientific and career development, along with the interrelation with his scientific peers at the time. I intend to delve further into these group relationships by looking at the development of Loeb’s research networks and the establishment of a new research style which became later taken up at the American Rockefeller Institute for Medical Research as well as a number of experimental research institutes in the Kaiser Wilhelm Society in Germany. Concerning the Rockefeller Foundation, Loeb was able to continue his laboratory studies that he began at the Naples marine research station through a number of personal research grants (Hollingsworth, 1986). They allowed him to regularly visit the Marine Biological Laboratory Woods Hole for his experimental research and to also teach courses from summer 1892 onward.

**FIGURE 5:**
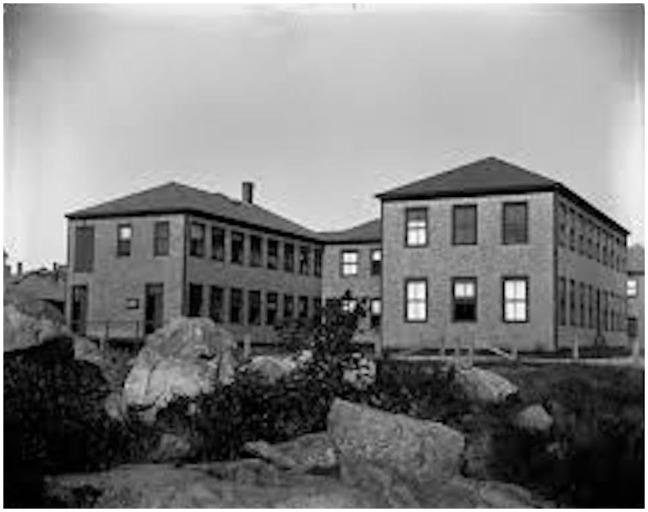
The Marine Biological Laboratory Woods Hole, Massachusetts, on the Atlantic Coast of the United States © Public Domain.

As German science historian Irmgard Mueller has shown, based on the files of the Rockefeller Archives, it is evident that Woods Hole (Figure 5) was intentionally modeled after the Naples marine research station. This is especially noticeable, according to Mueller, from the historical source of the proceedings papers of former board directors and their communication with science administrator Simon Flexner (1863–1946), who furbished suggestions, acquired resource materials, and acted as an important communicator and protagonist for the model of the interdisciplinary research stations (Mueller, 1987). At the beginning of the twentieth century, Flexner – supported by the Rockefeller Foundation and the Carnegie Foundation for the Advancement of Science – was put in charge of many large-scale projects to rearrange the medical and scientific institutions in the United States (Fosdick, 1989).

As Mueller has recently worked out, the “modernization” of Woods Hole also offered Loeb and other contemporary experimental biologists laboratory conditions that rivaled those previously found at the Mediterranean coast (Mueller, 2015).

“In America, Loeb was able to continue his Neapolitan studies on the physiology of development and on regeneration. From 1892 on, he regularly visited the Marine Biological Laboratory Woods Hole to do research and to give summer courses. Woods Hole had been modeled after the Naples Zoological Station and offered similar research conditions. Thus, Loeb could apply the methods he had learned in Europe in his new home. Loeb’s research on the physiology of development turned him into a well-known scientist among his contemporaries. Experimenting on the influence of inorganic substances on sea urchins’ eggs, he was able to publish his most famous finding: *On the Nature of the Process of Fertilization and the Artificial Production of Normal Larvae (Plutei) from the Unfertilised Eggs of the Sea Urchin* in 1899 (Loeb, 1899)” (Fangerau and Mueller, 2005, emphasis in the original).

On the other side of the Atlantic we find this systematically replicated in the corresponding archival material of the German Federal State Archives (“*Bundesarchiv*”) in Koblenz, demonstrating the influence of the German Research Council (“*Notgemeinschaft der Deutschen Wissenschaft / Deutsche Forschungsgemeinschaft*”), between 1920 and 1930 (Bundesarchiv, 1920–1930, Bestand R 73). It is necessary here to bring those research developments at the fringes of academic departments of anatomy, physiology, or neurology into focus too, since from the 1920s onward major research impulses developed out of city laboratories, private clinics, and institutes related to the Kaiser Wilhelm Society. These innovative work places were created apart from university settings – even though close collaborations ensued, when methodological and educational exchanges made interdisciplinary cooperation in the biology of the nervous system key to future scientific achievements (Stahnisch, 2009).

A major factor for the parallel growth of research institutions aside from established universities, hospitals, or medical practices was the availability of private philanthropic endeavors and effective research funding institutions, such as the University Foundation in Frankfurt am Main, the Krupp steel tycoon family’s support of scientific and medical research, along with Deutsche Forschungsgemeinschaft’s (“German Research Council”) actor-role after World War One (Stahnisch, 2019). These institutions operated even beyond national borders, very noticeable for example in the case of Loeb’s advisory role for the German Research Institute for Psychiatry in Munich (with its basic brain-research program) or The Rockefeller Foundation’s engagement in major German-speaking centers of the early brain sciences, in the wider regions of Munich, Berlin, Breslau, Leipzig, or Zurich (Engstrom et al., 2016).

The German Research Council particularly sought to establish interdisciplinary and international academic relationships – conceived as large-scale, nation-wide research programs (“*Gemeinschaftsaufgaben*”). These included socially relevant fields such as tumor research, the prevention of infectious diseases, and mental hygiene. This development became even more prominent when it came under the mandate of Prussian science administrator Friedrich Schmidt-Ott (1860–1956), the German Research Council’s inaugural president (Bundesarchiv, Bestand R 73, 1920). The tight cooperation and personal relationship was also visibly emphasized when Jacques Loeb passed away on February 11, 1924 on Bermuda, and Schmidt-Ott quickly took to sending his condolences to the Rockefeller Institute in New York – appreciating the enormous impact that Loeb exerted on the contemporary community of biologists working on the nervous system –, for which the American foundation was very grateful in their reply letter on February 24, 1924:

“My dear Sir:

In the absence of Mr. [George] Vincent [1864–1941] from New York permit me to thank for your kind letter of February 19[24] concerning Doctor Jacques Loeb.

Dr. Loeb was a member of staff of the Rockefeller Institute for Medical Research. I am therefore taking the liberty to refer your letter to the Director of the Institute, Doctor Simon Flexner, who, I know, will deeply appreciate your expressions of sympathy” (Bundesarchiv, Bestand R 73, 1924).

In the historical files documenting the financial support for the Kaiser Wilhelm Institute for Brain Research in Berlin or the German Research Institute for Psychiatry (Historical Archive of the Max Planck Institute for Psychiatry, 1929), the name of the Naples marine research station can be frequently found. It purchased equipment and infrastructure for the laboratories and assisted the upkeep of experimental technologies as the material basis for contemporary medical science and neurophysiological research (Pauly, 1987).

German physiologist Otto Heinrich Warburg (1883–1970) – Nobel prize laureate in Physiology or Medicine (1931) – who later became a leading science politician in the “shadow cabinets” of the Kaiser Wilhelm Society and German Research Council (Weisz, 2015), promoted the Loeb-Miklouho-Maclayian idea to organize more institutes for biological research in line with the Naples marine research station. With respect to research actions at the time, these need to be interpreted as constant forms of “interactions,” while establishing variable forms of agency, scientific ontology, flexible spaces, and with altering degrees of durability. Through these interactions – e.g., scientists traveling to the research stations, sea animals filling the aquaria for experimental work, and letters, papers, and specimens being sent internationally by railway and mail, etc., – Loeb’s experimental approaches became gradually taken up by a wider group of comparative neuroanatomists. They shared similar interests in neuronal degeneration and regeneration phenomena; *viz.* neuroanatomist Carl Weigert (1845–1904), neurophysiologist Albrecht Bethe (1872–1954) (Bethe, 1905), and Edinger at the Frankfurt Senckenberg Institute (Stahnisch, 2008).

“These two men [Ludwig Edinger in Germany and Clarence Luther Herrick in the United States] were generally regarded as the founders of comparative neurology as an organized scientific discipline, the one in Germany the other in the United States” (Contributions of the American Philosophical Society, 1955).

Bethe is quite interesting in this context, since he upheld durable contacts to Italian neurophysiologists and neuroanatomists, such as the experimental physiologist and regeneration researcher Filippo Botazzi (1867–1941). The latter chaired the physiology department of the Naples Zoological Station between 1915 and 1925, and Bethe referenced his work frequently in his own investigations on neuronal plasticity. He related, for example, that the size of fish, starfish, and mussels changed as a direct result of environmental milieus (Finger and Stein, 1982). According to Bethe, this observation could only be corroborated in experiments on the specific physico-chemical environments of such sea animals, despite the fact that the outcome of his experiments varied with the mucous membrane surfaces of these soft-skinned animals. Bethe himself used Aplysia as a frequent test animal for his plasticity research (Bethe, 1929), and his theoretical discussions of plasticity and neuroregeneration were themselves based on his previous marine experiments (Bethe and Fischer, 1931).

Furthermore, Edinger’s specific work on comparative neurology is worth mentioning, also because he engaged in a close correspondence with Loeb, after sending him his “*Twelve Lectures on the Structure of the Central Nervous System*” (1884) (Edinger, 1884), on the comparative approach in brain anatomy and physiology (Roux, 1899). Edinger had himself visited the Naples marine research station in 1904, which left a deep impression on him as well. Since Loeb saw that Edinger’s lectures, with their “modern pedagogical tendency,” were of interest to readers on the other side of the Atlantic, he asked his pupil Dr. Paul Carus (1852–1919) to translate Edinger’s lectures into English. A flattering review in the philosophical journal “*The Monist*” was consecutively published by Lyon (1897). With his own comparative approach to neurology, Edinger had promoted the relevance of Loeb’s findings and the potential impact on basic neuroanatomy and neurophysiology (Roux, 1900). In exchange, he nominated Loeb for the Soemmerring Prize in biology of the Frankfurt Senckenberg Foundation – and Loeb kept in contact and remained a member of the Senckenberg Natural History Society for many years (Senckenbergische Naturforschende Gesellschaft, 1912).

In a general correspondence to Edinger, dated December 24, 1896, Loeb had already outlined that his work was truly relevant for neurology in three specific regards: Firstly, he thought having elucidated instinct behavior in animals, through heliotropism, geotropism, and their related hereditary processes as a foundation of simple nervous reflexes. Secondly, under the influence of the French materialist philosophers following Henri de Saint-Simon (1760–1825) and the experimental physiologists around Charles-Édouard Brown-Séquard (1817–1894) (Stahnisch, 2003a), he understood it as one of his aims to provide a physico-chemical basis for the processes in mechanistic terms. This approach was also put forward in his works “*Ueber die Entstehung der Aktivitaetshypertrophie der Muskeln*” (Loeb, 1906b) or “*Ueber physiologischen Wirkungen des Sauerstoffmangels*” (Loeb, 1895). Thirdly, Loeb remained interested in the functional morphology of the Central Nervous System and brain development, inviting him later personally to come to the United States.

“Wouldn’t you like to come to America? You would be greeted here warmly. I have built myself a house very close to the university campus, and my wife and I will be delighted to receive yourself and your wife as our guests. I am really steadfast with this invitation, and look much forward to the day when you decide to travel to this side [of the Atlantic]. Also, you’ll be quite [interested] in reading that since [18]92 a third of the time of my Physiology course is dedicated to general and comparative physiology. This is the mandatory course for medical students; and, in addition, I teach brain physiology primarily from a comparative standpoint.

With warm greetings, I remain deeply dedicated, yours Jacques Loeb” (Loeb, 1896).

Together with Bethe (Bethe, 1933), Edinger often defended Loeb’s scientific approach in the German-speaking community against the neo-vitalist critique of zoologist Victor Franz (1883–1950) and his school, who regarded his biophysical ways to explain tropism as mere supposition. Conversely, Edinger’s high esteem for Loeb was represented in the major role that Loeb could assume during the process of finding a replacement for the position of the institute director after Edinger unexpectedly died (University Archives Frankfurt, 1918/19).

When Austrian physiologist Paul Weiss (1898–1989) from the University of Vienna applied for the directorship, it was Bethe who turned his application down. According to his assessment of March 20, 1918, in his articles Weiss had neglected Loeb’s ground-breaking experiments, such as those from 1902 that elucidated proposed cerebral memory mechanisms as a “tuning process” in sensory frequencies (Loeb, 1902). When thinking about the future of the “Edinger’s Institute” in Frankfurt am Main, Bethe was keen to move it into the administrative fold of the Kaiser Wilhelm Society to secure Edinger’s important legacy (Ibid.). As a precondition for its success, Bethe anticipated that the institute needed to open an experimental department aligned with Loeb’s categories to establish a more integrated program regarding the structure-function binary in brain research. He foresaw the incorporation of a comparative and zoological perspective into the workings of the Neurological Institute. However, this could not be realized due to the financial strains of the city of Frankfurt during the period of World War One, and its new university only was to open its doors in 1917 as a private institution.

With hindsight, it is remarkable how profound the projected value of Loeb’s research program was at the time. It included the advancement of existing institutions, as these should be integrated into a global network of zoological stations along the purported “railway network” model (Loeb, 1959). Loeb’s personal influence – and with it his career-forming experiences and learning from the Naples marine research station – can thus be seen to represent a new context of experimental neurological research during this time.

“He called attention to the work of investigators who had found that in the coloration of their skins certain fish reproduce a pattern, such as a checker board, forming the bottom of the aquarium. Extirpation of the eyes or of the optic ganglia in the brain or cutting the sympathetic nerve fibers which go to the pigmented cells of the skin prevents this phenomenon. Hence the path is known and Loeb suggested that what travels along this path may be an “image” in the sense that for each dark or bright point of the object there is a corresponding state of excitation first in the retina and subsequently in the optic nerves and in their terminal ganglia in the brain” (Osterhout, 1928).

Similar to Loeb, many neuroanatomists at the beginning of the twentieth century shifted their research focus to the cellular properties of neuronal de- and regeneration phenomena, a development which laid the basis for a new tradition in the history of neuroplasticity (DeFelipe and Jones, 1992). These new frontiers in the contemporary brain sciences, stimulated by the introduction of ever newer staining technologies for neurohistology, also gave rise to a better understanding of the morphological properties involved in neuroadaptive processes (Bethe, 1895). The gold-derivative staining method from neurohistologist Camillo Golgi (1843–1926) – himself visitor to Naples’ marine research station of 1919 – and methylene blue staining by German microbiologist Paul Ehrlich (1854–1915) can be named in this respect. Both stains were later used by Loeb and Bethe in their early research on “neuronal plasticity” until the 1930s (Bethe, 1930).

These staining techniques also gave rise to continued methodological discussions in contemporary nervous degeneration and regeneration research programs (Pannese, 1996). Siding with the non-localizationists of brain functioning, Jacques Loeb, understood the physiology of the organism in quite holistic terms of physiological interactions of individual neurons and the full complexity of their anatomical structures (Shepherd, 1991). This concept did not refer to the whole organism, yet to an interrelatedness of its parts that created the unity of each biological organism (Loeb, 1916). Modern biophilosophers have since come to use the concept of “emergent functions” to explain functional hierarchies in more intricate terms (Craver, 2007).

## Discussion

This article has looked at Jacques Loeb and his experiences at the Naples marine research station, along with his successive laboratory approaches at various institutes. Aspects that stimulated biological investigations in the fields of brain morphology have been singled out at the beginning of the twentieth century. It has become apparent that Loeb, who trained during the rise of modern scientific medicine, used biological processes themselves as models for human bodily and nervous system regeneration, as well as structural and functional processes of the brain’s organization in ontogenesis (Churchill, 2015). Taking Loeb’s experimental investigations here closer into focus, various philosophical, biological, and medical influences central to his work could be shown to resonate with the contingent contexts and resources of economic, organizational, and technical developments. Such resources were provided to him at the Naples marine research station, and later when working as a senior researcher in the biological research stations on the other side of the Atlantic. Yet when conducting his dislocated, though highly intriguing experiments in America in Woods Hole or at the Rockefeller Institute for Medical Research, Loeb continued to rely on and allude to his late-nineteenth century formative experiences at Naples – that in fact represented one of the most productive laboratory periods in his whole career with sixty publications appearing only in 3 years (Mueller, 1987).

Loeb’s reciprocal interest in the work of contemporary biologists working on the nervous system stemmed from multiple acquaintances with his peers, principally in the area of neuroregeneration research. Having pointed in this essay to his research program, institutional organizational arrangements were also important, such as the use and supply of experimental animals provided by the Naples marine research station for the investigation of basic neuroregeneration processes. As we have seen, such informal communication structures in the genteel atmosphere of Naples and in Goltz’s scientifically minded Strasburg institute – including broader networks of scientific exchanges with Edinger and his associates – helped integrating the various research localities into a functional whole, leading to new investigative styles and methods of neurophysiological research.

## Summary and Conclusion

Loeb’s line of reasoning on neuroplasticity was based on prolonged series of experimental research with marine animals, which he had pursued at the Italian marine laboratory before the First World War, along with findings that he later developed in his empirical work on the cortex in rabbits and dogs. These were squared with comparisons to human clinical and behavioral observations. When researching pathological and neurosurgical processes before and after the First World War, Loeb focused primarily on issues of nerve growth, biomechanics, and degeneration and regeneration phenomena in the central nervous system from a distinctly comparative perspective. Furthermore, Loeb’s contemporary research program supported the interdisciplinary formation, together with the neurobiological developments in the history of this young field from the 1910s to the 1930s (Klinke, 2006). In the context of neurohistological and neurophysiological work, many contemporary European neuroanatomists, neurophysiologists, and neurologists developed an interest in understanding the morphological properties, which the central nervous system possessed to react to degenerative disorders and injuries (Breidbach, 1997). Accordingly, neuroplasticity became a guiding leitmotiv in neurophysiological and neuroanatomical research about the genetic and restorative capacities of the human nervous system – as aspects that Loeb himself called “artificial life” (Loeb, 1913).

This article has sought to emphasize the paradigmatic changes that Loeb’s work brought about for early twentieth-century brain science. It explored various intellectual and organizational influences rooting in his early experiences at the Naples Zoological Station – this “Mekka for biologists of the whole world” (Juday, 1910) –, before Loeb became seen as the doyen of modern regenerative medicine, receiving multiple prizes and awards for his work, such as his four honorary degrees from Cambridge, Geneva, Leipzig, and Yale, his honorary membership of the Royal Belgian Academy of the Sciences and the Société de Biologie of Paris, as well as several nominations for the Nobel Prize too. His innovative interdisciplinary research style and epistemology thereby underwent a transformation following to his emigration. Loeb adapted an “American Style of Science” that had its origins in his previous European experiences before his emigration, acting as a catalyst for the implementation of “American style” biology back into the life sciences of Central Europe – together with his network of peers, collaborators, and students who shared many of his ideas.

Loeb’s research style later also influenced the make-up of a number of science units in the Kaiser Wilhelm Society and the Rockefeller Institute for Medical Research. It had been particularly visible at the “Institute for the Scientific Study of the Effects of Brain Injuries” at the University of Frankfurt am Main, where neurophysiologist Bethe continued Loeb’s successful research style to investigate phenomena of peripheral and central nervous system regeneration (Kobelt, 1930). In the latter part of his career, however, Loeb addressed more philosophical questions, including the history of experimental physiology, issues regarding the reorganization of medical and scientific education, along with epistemological foundations of brain research. These intellectual endeavors often followed from those stimulating discussions and exchanges that he had had at the Naples Zoological Station many decades before.

## Author Contributions

FWS carried out all the research, writing, formatting, and editing.

## Conflict of Interest Statement

The author declares that the research was conducted in the absence of any commercial or financial relationships that could be construed as a potential conflict of interest.
